# Comparative costs and potential affordability of a multifaceted intervention to improve treatment outcomes among people with HIV who inject drugs in Russia: economic evaluation of the LINC‐II randomized controlled trial

**DOI:** 10.1002/jia2.26208

**Published:** 2024-02-25

**Authors:** Sydney Rosen, Elena Blokhina, Ve Truong, Agata Bereznicka, Natalia Gnatienko, Emily Quinn, Dmitry Lioznov, Evgeny Krupitsky, Amy Michals, Karsten Lunze, Jeffrey H. Samet

**Affiliations:** ^1^ School of Public Health Boston University Boston Massachusetts USA; ^2^ Health Economics and Epidemiology Research Office Wits Health Consortium Faculty of Health Sciences University of the Witwatersrand Johannesburg South Africa; ^3^ Pavlov First State Medical University St. Petersburg Russian Federation; ^4^ Clinical Addiction Research and Education (CARE) Unit Section of General Internal Medicine Department of Medicine Boston Medical Center Boston Massachusetts USA; ^5^ Biostatistics and Epidemiology Data Analytics Center Boston University School of Public Health Boston Massachusetts USA; ^6^ Smorodintsev Research Institute of Influenza St. Petersburg Russian Federation; ^7^ V.M. Bekhterev National Medical Research Center for Psychiatry and Neurology Saint Petersburg Russian Federation; ^8^ Section of General Internal Medicine Department of Medicine Boston University School of Medicine Boston Massachusetts USA; ^9^ Department of Community Health Sciences Boston University School of Public Health Boston Massachusetts USA

**Keywords:** antiretroviral therapy, cost, cost‐effectiveness, injection drug use, opioid treatment, Russia

## Abstract

**Introduction:**

The LINC‐II randomized controlled trial in St. Petersburg, Russia for HIV‐positive adults who inject drugs found that a multi‐component intervention including initiation of antiretroviral therapy (ART) during admission to an addiction hospital, strengths‐based case management and naltrexone significantly increased 12‐month HIV viral suppression and ART retention. We conducted a comparative cost analysis to determine if the 12‐month cost of the intervention is affordable within the current Russian health system.

**Methods:**

We used LINC‐II trial records and questionnaire responses to calculate the resources utilized by each participant in the study, including inpatient days, medications, laboratory tests, outpatient consultations, case manager interactions and opioid medication treatment. Quantities of resources utilized were multiplied by unit costs for each resource estimated from the service fee or price lists used by the study facilities for each specific service delivered. We report the average cost/study primary (viral suppression at 12 months) or secondary (retention in care at 12 months) outcome/participant in 2021 USD and compare costs between study arms.

**Results:**

The trial enrolled 225 participants (111 intervention, 114 control) between September 2018 and December 2020. Viral suppression, non‐suppression and missing suppression results were 28% and 14%, 49% and 37%, and 31% and 41% for the control and intervention arms, respectively. Retention results were 35% and 51% for the control and intervention arms, respectively. The average cost per study participant was $2714 in the control arm and $4342 in the intervention arm. The average cost per participant virally suppressed at 12 months was $3662 (control) and $6355 (intervention). The average cost per participant retained at 12 months was $4050 (control) and $5448 (intervention). For those retained, the cost difference between the arms was comprised of opioid treatment (35%), case management (31%), outpatient visits (18%) and additional days of ART (12%).

**Conclusions:**

The LINC‐II intervention increased the cost of care for HIV‐positive people who inject drugs in Russia significantly, but some components of the intervention, particularly earlier initiation of ART and case management, may be justifiable due to their success in reaching a challenging subgroup of the population in need.

**Clinical Trial Number:**

NCT03290391

## 1 INTRODUCTION

HIV and injection drug use continue to impose heavy burdens of ill health on the Russian Federation (Russia). The World Health Organization reported that in 2021, HIV incidence was six times higher in Russia than in the rest of Europe [1]. HIV prevalence nationally was estimated at 0.68% in 2022 [2]. Uptake of antiretroviral therapy (ART) is reported to have increased sharply since 2018, to as high as 93% of people with HIV (PWH) who are diagnosed and recorded in the Federal HIV Register [2], though with a good deal of regional variation in coverage. Injection drug use remains a major risk factor for HIV acquisition in Russia [3, 4], and the proportion of people who inject drugs who are registered in the Federal HIV Register is likely relatively low.

In 2022, we completed a two‐arm, open‐label, individually randomized controlled trial of a multi‐component intervention intended to improve HIV treatment outcomes and increase opioid abstinence among PWH and injection drug use who were admitted to an addiction hospital in St. Petersburg, Russia [5], a city where HIV prevalence exceeds 0.9% [4]. The LINC‐II (Linking Infectious and Narcology Care‐Part II) trial observed significant improvements over standard of care in achieving the primary outcome of 12‐month HIV viral load suppression. Secondary outcomes also improved: rates of initiation of ART, retention in HIV care, antiretroviral medication coverage (possession of medications), and the composite outcome of undetectable HIV viral load and past 30‐day opioid abstinence at 12 months also increased significantly [6].

The LINC‐II trial intervention included three components: rapid access to ART, receipt of naltrexone for opioid use disorder, and 12 months of strengths‐based case management for PWH who inject drugs and were not on ART. HIV strengths‐based case management is an approach that uses a participant's strengths as the foundation to build capacities to engage in HIV medical care and improve HIV‐related health outcomes [6]. Eligible participants were enrolled upon admission to St. Petersburg's City Addiction Hospital. Unlike under standard of care, intervention arm participants were offered ART initiation, facilitated by an infectionist (HIV doctor), while still admitted to the addiction hospital, rather than being referred as outpatients to the separate City AIDS Centre for ART initiation after addiction hospital discharge. Participants were also offered one intramuscular gluteal injection of 380 mg of extended‐release naltrexone while admitted, followed by 1000 mg dissolvable naltrexone implants four times during the 12‐month follow‐up period. Finally, peer case managers who were PWH in recovery from opioid use disorder offered in‐person or phone‐based strengths‐based case management in up to 10 one‐on‐one sessions over that period. As we elaborate further below, intervention arm participants were significantly more likely to initiate ART, remain in care and achieve viral suppression than were control arm participants, with an adjusted odds ratio of 3.04 for achieving an undetectable viral load at 12 months.

As for any intervention that is effective in achieving the intended health outcomes, a key follow‐on question to answer is how much the intervention costs—that is, the average cost per outcome achieved, compared to the standard of care (cost‐saving)—and whether it is affordable within the health system. The LINC‐II intervention entailed new costs, such as the case managers and naltrexone implants, that are not incurred under standard of care. Despite this, LINC‐II may be a good investment because of the very large improvement observed in health outcomes. In this analysis, we estimate the average cost per participant enrolled in each arm of LINC‐II and each participant achieving the primary outcome of 12‐month viral suppression and a secondary outcome of 12‐month retention in care, compared to standard of care, and examine affordability within Russia's public healthcare system.

## 2 METHODS

For this analysis, we adapted micro‐costing methods developed for economic evaluations of HIV treatment in sub‐Saharan Africa [7, 8, 9]. As the LINC‐II protocol [5] and primary outcomes [6] have already been reported, we focus here on methods and study characteristics necessary to understand the cost analysis. Study procedures are illustrated in Figure S1.

### 2.1 Study population

LINC‐II took place at the City Addiction Hospital (CAH) in St. Petersburg, Russia, an inpatient hospital that provides addiction (narcology) care for those with substance use disorders. The trial enrolled adults who were admitted to CAH with the following characteristics: positive HIV status documented in their medical charts; not currently on ART (past 30 days); a history of injection drug use; and a current diagnosis of opioid use disorder.

### 2.2 Cost estimate methodology

Costs were estimated from the perspective of the healthcare provider, which for this trial included the City Addiction Hospital, the City AIDS Centre, the Pavlov State Medical University (where implants were provided) and local clinics, which offered chronic HIV care after ART initiation.

Individual participants’ utilization of resources was extracted from their medical records for HIV care and from study case reports for opioid care and case management over the 12‐month period from randomization to assessment of the primary outcome of viral suppression. Resources captured included inpatient days in the addiction hospital, inpatient days for HIV‐and TB‐related causes over the follow‐up period, outpatient visits to the City AIDS Centre, antiretroviral medications dispensed, laboratory tests conducted, and, for intervention arm participants, case manager visits, naltrexone injections and naltrexone implants provided. Quantities of each resource utilized were then multiplied by unit costs for each resource. Unit costs were estimated primarily from the service fee or price lists used by the study facilities for each specific service delivered (e.g. a bed day in the hospital, a specific type of lab test or a clinical consultation with a specific provider cadre). Fixed costs of the healthcare facilities, such as building space, utilities, equipment and administrative staff, were accounted for within these service fees set by the facilities and thus did not need to be added separately to our cost estimates.

### 2.3 Data collection

Data for the cost analysis were collected from three sources. First, we used routinely collected medical records from the City AIDS Centre to identify resources used for HIV care, such as antiretroviral medications, viral load tests and HIV‐related clinic visits. Second, study case report forms provided resource utilization data for the City Addiction Hospital, including number of days admitted and services provided; for study‐related resources used for the intervention arm, such as case manager visits and implants; and for participant‐reported resource utilization, such as additional hospitalizations during the follow‐up period. Third, unit costs were obtained from study sites’ price schedules, supplemented with study invoices and published information. Table 1 provides details on the types of data collected and the methods used for estimating each resource's unit cost.

**Table 1:** Resources included, data sources and methods for estimating unit costs of the LINC‐II trial study arms

Resource	Data source and method for estimating unit cost
Medications, devices and laboratory tests	Number of antiretroviral tablets and quantities of narcology medications and implants dispensed to study participants and types of laboratory investigations performed between study enrolment and the primary 12‐month endpoint were extracted from individual study records and clinic records for the intervention arm and from self‐report and clinic records for the control arm. For each medication and laboratory test, a unit cost was assigned based on the price schedule from the AIDS Centre (https://www.hiv‐spb.ru/lsn/price‐pay‐service.html), City Addiction Hospital (https://nhosp.ru/perechen‐platnyx‐uslug/) or Pavlov University (for naltrexone implants). Because we do not have data on actual antiretroviral regimens dispensed, all participants were assumed to receive a three‐drug antiretroviral regimen of lamivudine, tenofovir and efavirenz, which was the most common regimen in use during the study period and is the least expensive available. Laboratory tests that were done solely for research purposes and are not likely to be conducted if the intervention were adopted as standard care were excluded.
Inpatient days for narcology care	The number of days admitted to the City Addiction Hospital during the admission at which the participants were enrolled in the study was extracted from study case report forms. A standard cost/bed‐day was then applied using the official price schedule for the City Addiction Hospital.
Inpatient days for other care	The number of days admitted to other hospitals for HIV or tuberculosis care was self‐reported by participants. A standard cost/bed‐day was then applied, using those hospitals’ price schedules.
Specialist, outpatient and medication collection visits	Study participants had a number of different types of interactions with the healthcare system during enrolment and over the follow‐up period. These included consultation with an infectionist (HIV specialist doctor) at the addiction hospital, consultation with a narcologist at the addiction hospital, visits to the AIDS Centre or a local clinic to refill medication prescriptions and visits to the Pavlov University for naltrexone implants. The infectionist offered ART initiation to intervention arm participants, while control arm participants may have consulted the infectionist at the addiction hospital but were referred to the City AIDS Centre for ART initiation. The cost of each of these interactions was estimated from the price lists of the facilities.
Strengths‐based case manager costs	As case managers were an intervention‐specific resource, costs were calculated from study invoices and expenditure reports and estimated as an average cost per session conducted, stratified by type of session (baseline, in‐person, telephone). Cost estimates included case manager salaries, transport, telephone and message fees, and refreshments. (Every patient in the intervention arm was assigned a case manager, but the number of case management sessions conducted varied widely by participant, up to a maximum of 10.)

Abbreviation: ART, antiretroviral therapy.

### 2.4 Outcomes and data analysis

To ensure that this analysis would be useful to local policymakers and implementers as well as other researchers, we estimated several cost outcomes, each stratified by study arm, as shown in Table 2. The primary outcome from the clinical trial, viral suppression (<40 copies per ml) at 12 months of study enrolment, was used as the primary measure of effectiveness for this analysis as well. Because many participants were missing 12‐month viral load test results, however, we added a third group to the primary outcome analysis, those with unknown viral suppression status. In a secondary analysis, participants were stratified into three groups that more closely reflect anticipated resource utilization: (1) initiated ART within 28 days of study enrolment and virally suppressed at 12 months; (2) did not initiate ART within 28 days; and (3) initiated within 28 days but did not have evidence of viral suppression at 12 months. For both primary and secondary outcomes, we used the viral load test result closest to the 12‐month endpoint, within a 90‐day window.

**Table 2:** Outcomes estimated for the cost and cost‐effectiveness analysis of the LINC‐II trial

Outcome	Method for estimating cost/outcome
Cost/patient served	Total costs in study period/total number of participants enrolled, by study arm. This estimate is useful for budgeting purposes, as the difference between the arms indicates the additional budgetary resources needed to implement the intervention per patient served.
Cost/primary outcome	Total costs for patients stratified by trial primary outcome (virally suppressed at 12 months; not suppressed at 12 months; no viral load test result available at 12 months) and arm. The difference in cost between outcomes indicates the anticipated difference in budgetary needs if viral suppression rates improve.
Total cost/patient suppressed	Total cost from enrolment to 12 months for all patients in the arm/total number of patients in the arm achieving the primary outcome of 12‐month viral suppression. This estimate focuses on cost differences between the arms for patients who do achieve the primary outcome. It takes into account all costs incurred for all patients but divides by only the number with a successful outcome, and thereby links the cost of service delivery to the primary outcome.
Cost/secondary outcome	Total costs for patients stratified by a secondary outcome (initiated ART within 28 days and retained at 12 months; did not initiate ART within 28 days; initiated ART within 28 days but not retained at 12 months). The difference in cost between outcomes indicates the anticipated difference in budgetary needs if retention outcomes improve.
Cost‐effectiveness	Although we did not undertake a full cost‐effectiveness analysis, we did estimate the incremental cost‐effectiveness ratio (ICER) = difference in costs (intervention arm‐control arm)/difference in outcomes (intervention arm‐control arm). The ICER indicates the incremental additional cost to achieve one additional successful outcome. We estimated ICERS for both the primary and secondary outcomes defined above.

Abbreviations: ART, antiretroviral therapy; ICER, incremental cost‐effectiveness ratio.

We estimated average, undiscounted costs in 2021 U.S. dollars with 95% confidence intervals. Russian rubles were converted to USD at the average 2021 exchange rate of 70.92/$1.

Partial cost‐effectiveness of the intervention was estimated with an incremental cost‐effectiveness ratio (ICER), calculated as the additional cost to achieve one additional participant who initiated ART within 28 days and remained in care at 12 months (secondary outcome). We chose the secondary, rather than primary, outcome for the cost‐effectiveness analysis due to the large proportion of missing data in determining primary outcomes. Because of the relatively short follow‐up period of the study (12 months), lack of published information about long‐term retention on ART in this population, and overall low rates of viral suppression and 12‐month retention achieved in either arm, we opted not to estimate cost per life‐year gained or per utility metric. Our analysis reflects a partial cost‐effectiveness ratio because we did not incorporate potential long‐term cost savings attributable to avoided care for untreated HIV and to potential reductions in HIV transmission as a result of the intervention.

Finally, we evaluated the cost of the LINC‐II intervention in the context of existing healthcare spending and GDP/capita for Russia, to help inform policymakers about the affordability and priority of scaling up the programme.

### 2.5 Ethical review and role of funding source

The institutional review boards of Boston University Medical Campus and Pavlov University approved the LINC‐II study, including this cost analysis. The study is registered with ClinicalTrials.gov through the National Institutes of Health (NCT03290391). All participants provided written informed consent. The funder of the study had no role in study design, data collection, data analysis, data interpretation or writing of the report.

## 3 RESULTS

### 3.1 Clinical trial outcomes

The trial enrolled a total of 225 participants from inpatient wards at the City Addiction Hospital in St. Petersburg, Russia between 19 September 2018 and 25 December 2020. Follow‐up was completed in March 2022. The analytic sample included 111 in the intervention arm and 114 in the control arm. Participant characteristics are reported in Table S1.

At 12 months after randomization, 47% of the intervention group and 23% of the control group had an undetectable HIV viral load (<40 copies per ml) (adjusted odds ratio [AOR] 3.04; 95% confidence interval [CI]: 1.44, 6.44; *p* = 0.004) based on a multiple imputation analysis [6]. For this cost‐effectiveness analysis, as mentioned above, we assigned those with missing viral load test results to their own analytic group, rather than imputing outcomes. For our analysis, 28% of the intervention arm and 14% of the control arm had documented viral suppression; results for documented non‐suppression and missing suppression data were 49% and 37% for the control arm and 31% and 41% for the intervention arm, respectively.

For the secondary outcome, those assigned to the LINC‐II intervention had substantially higher odds of initiating ART within 28 days of randomization (74% vs. 11% for intervention and control groups, respectively). Intervention participants also had higher odds of remaining in HIV care (51% vs. 35%), defined as ≥1 clinic visit in two consecutive 6‐month periods, as determined by medical record review. Average ART coverage over the study period, defined as the number of days of ART medication dispensed over the total number of days in the study, was 41% for the intervention participants and 22% for controls [6].

### 3.2 Resource utilization and unit costs

Average resource utilization by study arm is shown in Table 3. As might be expected, participants in the intervention arm spent more time on ART and thus utilized more antiretroviral medication during the study period, as most initiated much sooner after study enrolment than did control arm participants (average of 30 days after enrolment vs. 122 days after enrolment, for those who initiated ART during the study period). Intervention arm participants also utilized a number of resources required for the intervention itself, such as case manager visits and naltrexone. Intervention arm participants spent slightly longer (average of 1.6 days) in inpatient care at the City Addiction Hospital, a difference that could be associated with the time required for intervention procedures.

**Table 3:** Average resource utilization per participant in the LINC‐II trial, by study arm, difference in quantities utilized between arms and unit cost per resource

				Unit cost		
Resource*^a^*	Control arm average quantity used/participant *N* = 114	Intervention arm average quantity used/participant *N* = 111	Difference in average quantity used/participant (intervention‐control)	Unit	Cost/unit (₽, rubles)	Cost/unit (USD 2022)
*Inpatient and outpatient care*						
Inpatient days, City Addiction Hospital	15.9	17.5	1.6	Day	₽7887	$111.21
Inpatient days, HIV hospital	3.0	3.1	0.1	Day	₽11,200	$157.92
Case management visits—baseline (int)	0.0	1.0	1.0	Visit	₽4140.00	$58.38
Case management visits—in person (int)	0.0	3.9	3.9	Visit	₽4140.00	$58.38
Case management visits—phone (int)	0.0	2.4	2.4	Visit	₽3140.00	$44.28
Narcologist visits (City Addition Hospital)	0.2	0.1	‐0.1	Visit	₽1000	$14.10
Infectionist visit (City Addition Hospital)	0.8	0.9	0.1	Visit	₽1000	$14.10
Psychiatrist visit (City AIDS Centre)	0.1	0.0	0.1	Visit	₽1800	$25.38
Infectionist visit (City AIDS Centre)	3.6	4.0	0.4	Visit	₽1800	$25.38
ART refill visits (City AIDS Centre or local clinic)	1.9	3.7	1.8	Visit	₽300	$4.23
Laboratory test visit (City AIDS Centre)	0.6	0.6	0.0	Visit	₽260	$3.67
Implant visits and implant medication checkups (Pavlov University) (int)	0.0	3.4	3.4	Visit	₽3900	$54.99
Suture removal visits (Pavlov University (int)	0.0	1.5	1.5	Visit	₽750	$10.58
Suture removal visits with adverse events (Pavlov University) (int)	0.0	0.0	0.0	Visit	₽2000	$28.20
*Medications and lab tests*						
ARVs dispensed (tablets)^b^	403	856	453	Tablet	₽33	$0.46
Naltrexone (Vivitrol) injections (int)	0.0	0.6	0.6	Injection	₽19,935	$281.09
Naltrexone implants (int)	0.0	1.0	1.0	Implant	₽16,000	$225.61
Naloxone tests (int)	0.0	1.5	1.5	Test	₽680	$9.59
ALT/AST tests	0.9	2.0	1.1	Test	₽490	$6.90
CBC tests	0.9	1.1	0.2	Test	₽720	$10.15
CD4 counts	0.9	1.1	0.2	Test	₽2200	$31.02
HIV viral load tests	1.1	1.3	0.2	Test	₽6100	$86.01
Syphilis tests	0.4	0.3	‐0.1	Test	₽1030	$14.52
HCV tests	0.0	0.1	0.1	Test	₽550	$7.76
Chest X‐rays	0.2	0.2	0.0	Test	₽680	$9.59
Ultrasounds	0.0	0.0	0.0	Test	₽2700	$38.07
Pregnancy tests	0.1	1.3	1.2	Test	₽134	$1.89
Urine toxicology tests	0.3	3.2	2.9	Test	₽671	$9.46

Abbreviations: ALT/AST, aspartate transaminase/alanine transaminase; ARVs, antiretroviral medications; CBC, complete blood count; HCV; hepatitis C; USD, United States dollars.

^a^ Resources used only for the intervention are labelled “int.”

^b^ Each patient is assumed to require 3 tablets per day, one for each antiretroviral medication in the standard regimen. A 12‐month supply is thus 12 months x 30 days/month x 3 tablets/day = 1080 tablets.

As is evident from Table 3, uptake of the three components of the LINC‐II intervention in the intervention arm varied, with associated implications for resource utilization. Just over half (63/111, 57%) of intervention arm participants received a naltrexone injection at baseline and a tenth (10/111, 9%) received all four implants during follow‐up. All intervention participants attended at least one strengths‐based case management session, 83/111 (75%) attended at least five sessions and 55/111 (50%) attended all 10 sessions.

### 3.3 Cost per participant and cost per outcome

The average cost per participant by study arm and patient outcome is reported in Table 4. Independent of outcome, the cost per participant enrolled was 60% more ($1628) in the intervention arm than in the control arm. For those with documented viral suppression at 12 months, costs in the intervention arm were substantially higher, averaging almost $2700 more than in the control arm, an increase of 74% over the control arm average cost/participant. Differences in costs between arms were somewhat more modest for participants who were unsuppressed or of unknown viral suppression status. A similar pattern prevailed for the secondary outcome of retention in care at 12 months, though the differences between arms were smaller. For those who initiated ART and remained in care at 12 months, the intervention arm cost just 30% more than the control arm per participant.

**Table 4:** Cost per LINC‐II trial participant by study outcome and cost breakdown by resource utilized

Cost per participant	USD costs (95% confidence interval)				
	Control arm		Intervention arm		Difference between arms
	*N*	Cost	*N*	Cost	
All participants	114	$2714	111	$4342	$1628
***Primary outcome***					
Virally suppressed at 12 months	16	$3662 ($2754−$4569)	31	$6355 ($3850−$8860)	$2693
Virally unsuppressed at 12 months	56	$2873 ($2269−$3478)	34	$3945 ($3314−$4575)	$1072
Unknown viral suppression status	42	$2081 ($1737−$2424)	46	$3186 ($2663−$3708)	$1105
***Secondary outcome***					
Initiated ART ≤28 days and retained at 12 months	40	$4050 ($3271−$4828)	54	$5448 ($3961−$6935)	$1398
Did not initiate ART ≤28 days	57	$1784 ($1550−$2018)	18	$2764 ($2000−$3528)	$980
Initiated ART ≤28 days but not retained at 12 months	17	$2543 ($2049− $3036)	39	$3429 ($2830−$4029	$886

*Participants achieving secondary outcome of retention in care at 12 months (n = 40)*					
Resource	Control arm		Intervention arm		Difference between arms (% of difference)
	Cost	% of total	Cost	% of total	
ARVs	$435	11%	$603	11%	$168
Opioid treatment^a^	$0	0%	$489	9%	$489
Case manager interactions	$0	0%	$439	8%	$429
Laboratory tests	$256	6%	$322	6%	$66
Inpatient bed days	$3149	78%	$3131	57%	−$18
Clinic visits and consultations^b^	$210	5%	$462	8%	$252
Total per patient achieving outcome	$4050	100%	$5448	100%	$1398

Abbreviations: ART, antiretroviral therapy; ARVs, antiretroviral medications; USD, United States dollars.

^a^ ncludes naltrexone injections and implants (intervention arm only).

^b^ Includes consultations while admitted to narcology or other hospitals (both study arms).

Table 4 also reports the cost breakdown for study participants who achieved the secondary outcome of initiation within 28 days and retention at 12 months. As expected, antiretroviral medications for the 12‐month period cost more for intervention arm participants because they spent more days on treatment. The cost of opioid treatment (naltrexone tests and implants) was incurred only by intervention arm patients, adding substantially to the intervention arm total. Intervention arm participants also incurred substantial costs for case manager interactions and somewhat more than control arm participants for clinic visits. Differences between arms for laboratory tests and inpatient bed days were modest.

### 3.4 Cost‐effectiveness and affordability

Using the results shown in Table 4, we estimated an ICER of $12,199 per additional participant achieving the secondary outcome of ART initiation within 28 days of study enrolment and retention in care at 12 months. As mentioned above, this estimate does not take into account cost savings that may occur as a result of the intervention due to avoided complications and medical care of HIV disease and HIV transmission from intervention arm participants. Most of these benefits would only be realized in the long‐term, beyond the 12‐month follow‐up period of this study.

As is suggested in the primary outcomes manuscript for this trial, the opioid treatment component of the LINC‐II intervention may not have been essential to achieving HIV‐related outcomes, such as viral suppression, as uptake of the naltrexone implants was very low, with fewer than 10% of the cohort receiving all four quarterly implants. This is less likely to be the case for the case manager interactions; three‐quarters of the cohort received at least five case management sessions. If we estimate cost‐effectiveness excluding opioid treatment costs, the additional cost per additional participant achieving the secondary outcome falls to $8947.

The affordability of the LINC‐II intervention within the Russian healthcare system can be explored by considering Russia's GDP/capita and healthcare spending. Russia's 2021 GDP/capita was $12,195 [10], almost identical to the ICER estimated for LINC‐II. (We emphasize that our ICER is *not* for a life year gained, quality‐adjusted life‐year gained or disability‐adjusted life‐year averted; a GDP/capita threshold cannot thus be used to claim cost‐effectiveness but does provide some context for intervention costs.) In 2020, annual healthcare spending per person in Russia averaged $774 [11]. Based on the unit costs of resources shown in Table 3 and resource utilization data in Table 4, we estimate that the cost of HIV treatment alone for our study population is roughly $1387/year, a total that includes ARV medications, laboratory tests and outpatient consultations but no inpatient care.

## 4 DISCUSSION

The LINC‐II intervention, which combined rapid HIV treatment initiation, strengths‐based case management and opioid medication treatment, significantly improved HIV viral suppression and retention in care at 12 months after ART initiation. To achieve this success, it utilized several additional resources beyond those required for standard of care, including case managers not currently employed by the healthcare system and the provision of naltrexone implants. It also led to substantially earlier initiation of ART and resulted in participants receiving many more months of antiretroviral medications. Each of these resources was expensive, making it important to estimate the cost‐effectiveness of the intervention, and not just its health outcomes.

In this cost analysis, we found that 1 year of services for participants in the LINC‐II intervention arm cost an average of $1628, an amount that is 60% more than 1 year of services for participants in the control arm. The higher costs in the intervention arm were comprised largely of the opioid medication treatment and the case manager salaries, which were not offered in standard of care. Because intervention arm patients were on ART longer, they also incurred higher costs for clinic visits and medications. This is an expected outcome of interventions that improve retention in care: patients who are retained cost more than those who are lost. These additional costs may well be offset, however, by a reduction in the need for acute care among untreated patients whose HIV leads to severe illness later on. We note that scaling up the intervention may reduce the cost/participant slightly, for example by making more efficient use of case managers’ time. Most of the costs incurred, however, were variable (per participant) and will not change substantially with scale.

A number of studies have estimated HIV and/or opioid use disorder treatment costs in Russia and its neighbouring states [12, 13, 14, 15, 16, 17]. Most of these, however, use cost estimates from other countries, such as Vietnam [15] or Canada [13], and/or rely on data that are now many years out of date [14]. A modelling exercise for Eastern Europe (excluding Russia) reported a 2018 unit cost of $1438/patient/year in Kazakhstan, which has the GDP/capita closest to Russia's among the countries studied [12]. Another report indicates an average HIV treatment cost in 2021 of $1133/patient/year for migrants in Russia who do not have access to public services [16]. As noted above, in our data, combining antiretroviral medications, laboratory tests, and outpatient clinic visits and consultations, we estimated an average cost of $1387/patient/12‐month period in the intervention arm, consistent with prior estimates for HIV treatment alone.

The affordability of the LINC‐II intervention for the Russian healthcare system will depend in part on how the package of three components is implemented. Initiating ART while eligible patients are admitted to the hospital for treatment of opioid use disorder is likely to be very close to cost‐neutral in terms of services provided; the main additional expense of this component of LINC‐II will be additional months of antiretroviral medications and treatment monitoring required for individuals who start ART earlier, which we estimated to average $420/participant (Table 4; sum of differences for ARVs + clinic visits and consultations). Since initiating ART as soon as possible after HIV acquisition has multiple benefits and is consistent with Russian HIV guidelines, this component should comprise a justifiable expense within the healthcare budget.

The other components of the LINC II intervention—case managers and opioid treatment—each increased the estimated cost/participant by $400−500, or just under 10% of the total cost/participant (Table 4). These expenditures may be more difficult to justify in the context of a healthcare system that spends approximately $774/capita on healthcare (2020 estimate) [11]. The case managers may be a critical input into the positive outcomes achieved in the LINC‐II trial, however, and thus may be a valuable use of additional resources [17]. The low uptake of opioid treatment in the intervention arm, and its unclear contribution to outcomes observed, may make this component appear less affordable within Russia's healthcare budget. Policymakers may thus find it useful to consider each component of the intervention separately, rather than treating it as a single indivisible package. In the future, assessment of the outcomes of the selected components could be evaluated to confirm our speculation about the relative effectiveness of each component.

This cost analysis of the LINC‐II intervention had a number of limitations. It is a single‐site study of patients cared for in specialized facilities in a relatively well‐resourced urban setting. Much of the data collection for LINC‐II took place during the COVID‐19 pandemic. Restrictions on movement and individuals’ fear of visiting clinics during this period may have reduced healthcare utilization, which would also have suppressed treatment costs. In the absence of COVID‐19 restrictions and fears, uptake of ART in the control arm of LINC‐II may have increased; on the other hand, outcomes among those who did initiate ART, most of whom were in the intervention arm, may also have improved. In addition, as mentioned in the introduction above, uptake of ART in the general population rose sharply over the COVID‐19 era. We do not know if uptake in the LINC‐II study population mirrored these changes but suspect that uptake among people who inject drugs remains low.

We assumed that all participants were prescribed the same low‐cost ARV regimen; if some were instead given dolutegravir, which is gradually replacing efavirenz in Russia, medication costs for those individuals would be substantially higher. We excluded opportunity and direct costs to study participants, for example for missed work time or transport fares. Because the LINC‐II intervention required additional healthcare system interactions, costs to participants were likely higher in the intervention arm, though participants for whom the intervention was successful in achieving viral suppression likely saved substantial costs for HIV‐related healthcare in the long‐term. Finally, as noted above, the analysis excluded potential savings due to reductions in HIV‐related healthcare costs and HIV transmission. Incorporating these longer‐term savings would make the LINC‐II intervention considerably more attractive from a relative cost standpoint.

## 5 CONCLUSIONS

The LINC‐II intervention, which significantly improved uptake of HIV treatment and HIV treatment outcomes for a difficult‐to‐reach population of people who inject drugs in Russia, incurred a relatively high total cost per individual served and per positive outcome achieved. Each component of the intervention, however, came at a different cost. The cost of the two components that appear most likely to have contributed to the positive outcomes—ART initiation during inpatient admission at the narcology hospital and active support from case managers—may be affordable and an important strategy for achieving Russia's HIV programme goals.

## COMPETING INTERESTS

The authors declare that there are no competing interests.

## AUTHORS’ CONTRIBUTIONS

JHS, KL, AR, SR and EK designed the LINC‐II study and secured funding. SR conducted this analysis and wrote the first draft of the manuscript. NG and VT participated in the study design and study implementation and contributed to this analysis. EQ and AM carried out statistical analyses. EB, EK, NB and DL led study implementation in the field. EB contributed to this analysis. All authors critically reviewed and provided feedback on the article.

## FUNDING

This study was supported by a grant from the National Institute on Drug Abuse (R01DA045547) and the Providence/Boston Center for AIDS Research (P30AI042853).

## Supporting information

Supporting Information

Supporting Information

## Data Availability

Data collected for the LINC‐II study are available to interested investigators in the URBAN ARCH Repository: www.urbanarch.org. All cost data used in this analysis are reported in the manuscript.
