# Supplementary figures and images for: Comparative costs and potential affordability of a multifaceted intervention to improve treatment outcomes among people with HIV who inject drugs in Russia: economic evaluation of the LINC‐II randomized controlled trial
Source: J Int AIDS Soc. 2024 Feb 25;27(2):e26208. doi: 10.1002/jia2.26208 (PMC10895073; doi:10.1002/jia2.26208)

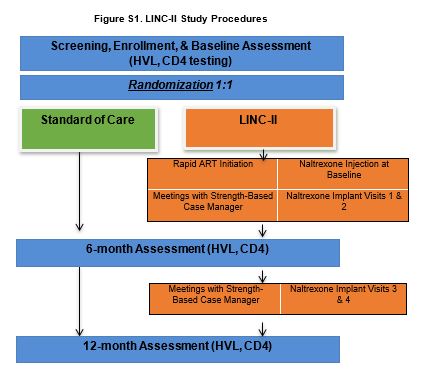

Supplement: Supplementary file 1 — Supporting Information [file JIA2-27-e26208-s002.JPG]
