# Supplementary material for: Comparative costs and potential affordability of a multifaceted intervention to improve treatment outcomes among people with HIV who inject drugs in Russia: economic evaluation of the LINC‐II randomized controlled trial
Source: J Int AIDS Soc. 2024 Feb 25;27(2):e26208. doi: 10.1002/jia2.26208 (PMC10895073; doi:10.1002/jia2.26208)
Supplement: Supplementary file 2 — Supporting Information [file JIA2-27-e26208-s001.docx]

**Table 1. Baseline Characteristics of LINC-II Study Participants - PWH Who Inject Drugs in St. Petersburg, Russia (2018-2020)**

| Demographics | Standard of Care  (n=114) | LINC-II Intervention  (n=111) |
| --- | --- | --- |
| Male  Female | 72 (63·2%)  42 (36·8%) | 64 (57·5%)  47 (42·5%) |
| Race  White  Asian  Other | 113 (99·1%)  1 (0·9%)  0 (0·0%) | 110 (99·1%)  0 (0·0%)  1 (0·9%) |
| Age (years), mean (sd) | 37 (5) | 37 (5) |
| Married, living with a partner, or in long-term relationship | 67 (58·8%) | 67 (60·4%) |
| Partner living with HIV | 30 (26·3%) | 28 (25·2%) |
| Education > 9 grades | 109 (95·6%) | 107 (96·4%) |
| Employed full time | 41 (36·0%) | 28 (25·2%) |
| Monthly income ≤20,000 rubles (~$290) | 31 (27·2%) | 37 (33·3%) |
| More than 1 person in household (does not live alone) | 104 (91·2%) | 101 (91·0%) |
| Did not run out of money for housing/food (past 12 months) | 56 (49·1%) | 45 (40·5%) |
| Arrested (ever) | 110 (96·5%) | 110 (99·1%) |
| Jail or prison (prior to past year) | 74 (67·3%) | 77 (70·0%) |
| Social Support Scale  Above median | 55 (48·2%) | 56 (50·5%) |
| Stable living quarters (past month) | 106 (93·0%) | 103 (92·8%) |
| Health Characteristics |  |  |
| CD4 cell count (cells/mm3), mean (sd) | 426 (282) | 415 (293) |
| Lowest CD4 cell count (self-report), mean (sd) | 339 (248) | 390 (217) |
| Viral load suppression (<40 copies per mL) at baseline | 4 (4·0%) | 4 (3·8%) |
| Years since initial positive HIV test, mean (sd) | 10 (6) | 11 (7) |
| Ever ART use* | 38 (33·3%) | 37 (33·3%) |
| Receipt of medication treatment for alcohol use disorder (ever) | 1 (0·9%) | 1 (0·9%) |
| Receipt of medication treatment for opioid use disorder (ever) | 5 (4·4%) | 13 (11·7%) |
| Most recent outpatient HIV care visit  More than a year ago  In the last year | 34 (29·8%)  44 (38·6%) | 35 (31·5%)  48 (43·2%) |
| Depressive symptoms^a^ | 89 (78·1%) | 83 (74·8%) |
| HCV (self-report) | 103 (90·4%) | 104 (93·7%) |
| Receipt of HCV treatment (ever) | 6 (5·3%) | 3 (2·7%) |
| Barriers to Medical Care, mean (sd)^b^ | 2 (2) | 3 (2) |
| GAD-7 Anxiety Score  Minimal anxiety  Mild anxiety  Moderate anxiety  Severe anxiety | 26 (22·8%)  44 (38·6%)  24 (21·1%)  20 (17·5%) | 28 (25·2%)  24 (21·6%)  31 (27·9%)  28 (25·2%) |
| HIV Stigma Score, mean (sd)^c^ | 2·3 (0·6) | 2·2 (0·6) |
| Self-reported Health  Fair/poor  Excellent/very good/ good | 73 (64·0%)  41 (36·0%) | 80 (72·1%)  31 (27·9%) |
| Substance Use |  |  |
| Alcohol Use: AUDIT  Abstinent or lower risk drinking  Hazardous use  Harmful use  Possible use disorder | 63 (55·8%)  13 (11·5%)  14 (12·4%)  23 (20·4%) | 53 (49·5%)  21 (19·6%)  7 (6·5%)  26 (24·3%) |
| Regular smoker^d^ | 109 (95·6%) | 111 (100·0%) |
| Opioid Use Disorder Severity  No OUD  Moderate  Severe | 1 (0·9%)  2 (1·8%)  111 (97·4%) | 0 (0·0%)  0 (0·0%)  111 (100·0%) |
| Current marijuana use^e^ | 30 (26·3%) | 22 (19·8%) |
| Shared works (past 30 days prior to hospitalization) | 51 (46·8%) | 51 (48·1%) |

^a^ Based on CES-D score of 16 or greater

^b^ Range 0-11

^c^ 10-40 - Berger HIV Stigma Scale

^d^ Smoking at least one cigarette per day

^e^ Past 30 days prior to hospitalization

* Randomization was stratified by history of ART use
